# Factors Associated With Non-Uptake of Implantable Cardioverter-Defibrillator (ICD) Among Eligible Patients at a Tertiary Hospital in Kenya

**DOI:** 10.5334/gh.1346

**Published:** 2024-08-16

**Authors:** Emmanuel Oluoch, Jasmit Shah, Mohamed Varwani, Mohamed Jeilan, Mzee Ngunga

**Affiliations:** 1 Department of Internal Medicine, Aga Khan University, Nairobi, Kenya; 2 Brain and Mind Institute, Aga Khan University, Nairobi, Kenya

**Keywords:** Implantable Cardioverter-Defibrillator (ICD), non-uptake, Sub-Saharan Africa (SSA)

## Abstract

**Background:**

Efficacy of Implantable Cardioverter-Defibrillator (ICD) implantation in both primary and secondary prevention of Sudden Cardiac Death (SCD) in at-risk population is well established. ICD implantation rates remain low particularly in Africa with a paucity of data regarding factors associated with non-uptake.

**Objectives:**

The primary study objective was to determine the factors associated with non-uptake of ICD among heart failure (HF) patients with reduced ejection fraction (EF<35%). Reasons for ICD refusal among eligible patients were reviewed as a secondary objective.

**Methods:**

This was a retrospective study among HF patients eligible for ICD implantation evaluated between 2018 to 2020. Comparison between ICD recipient and non-recipient categories was made to establish determinants of non-uptake.

**Results:**

Of 206 eligible patients, only 69 (33.5%) had an ICD. Factors independently associated with non-uptake were lack of private insurance (42.3% vs 63.8%; p = 0.005), non-cardiology physician (16.1% vs 5.8%; p = 0.045) and non-ischemic cardiomyopathy (54.7% vs 36.4% p = 0.014). The most common (75%) reason for ICD refusal was inability to pay for the device.

**Conclusion:**

ICDs are underutilized among eligible HF with reduced EF patients in Kenya. The majority of patients without ICD had no private insurance, had non-ischemic cardiomyopathy and non-cardiology primary physician. Early referral of HF with reduced EF patients to HF specialists to optimize guideline-directed medical therapy and make ICD recommendation is needed.

## Introduction

Sudden Cardiac Death (SCD) is a major cause of mortality among patients with cardiovascular disease accounting for about 50% of deaths (1). The majority of cardiac arrests are caused by ventricular arrhythmias in the background of structural heart disease often precipitated by an acute coronary event (2).

Implantable Cardioverter-Defibrillator (ICD) is recommended in individuals who are at a high risk of sudden arrhythmic death. Multiple trials have established a mortality benefit of this device therapy among survivors of life-threatening ventricular arrhythmia (3). Similarly, ICDs have been proven to lessen mortality in at-risk individuals as primary prevention (4,5,6).

Despite current practice guidelines and recommendations for ICD use among eligible patients, the utilization of ICD remains low particularly in Africa (6,7,8,9). Factors associated with non- uptake of ICD implantation are heterogeneous and include physician, patient and healthcare system factors (6,8,9,10). This study aimed to characterize these factors and identify reasons for ICD refusal among eligible heart failure (HF) with reduced ejection fraction (EF < 35%) patients at a tertiary hospital in Kenya.

The medical landscape in Kenya is rather unique with hardly any ICDs being performed in the public sector, due to a multitude of factors. In view of this, the expertise for the implantation and care of ICDs is confined to the private sector where almost all ICDs are implanted.

## Methods

### Study design, setting and population

This retrospective study was conducted at the Aga Khan University Hospital, Nairobi (AKUHN), between January 2018 and December 2020. AKUHN is a tertiary healthcare facility in Kenya. The hospital has countrywide referrals in addition to referrals from neighboring countries including Uganda, Rwanda, Tanzania, Congo and South Sudan. Referral process is twofold. Firstly, direct patient referral by their primary physicians. Secondly, through a dedicated patient referral office which links patients to care. This site was selected because it serves as a referral center in the SSA region for patients with severe HF for etiological evaluation and management including device therapy.

The study participants were HF patients with reduced left ventricular ejection fraction (LVEF ≤ 35%) on echocardiographic assessment and age >18 years. Exclusion criteria were: presence of ICD or CRT-D, improved LVEF to >35% after at least three months of follow up and those in NYHA IV functional class who are not candidates of CRT, Left Ventricular Assist Device and cardiac transplantation with an expected life expectancy of less than a year.

The study was approved by the Institutional Ethics Review Committee at AKUHN before conducting the study (2021/IERC 145 v1).

### Measurements

Baseline data was collected by a predefined case report form (Supplementary file 1). The medical case notes were assessed for (1) demographic data; age, sex, ethnicity and residence, (2) clinical factors; etiology of heart failure, NYHA class, history of syncope, ventricular arrhythmias, comorbidities, baseline electrocardiogram (ECG), (3) hospital factors; patient’s primary physician and mode of payment of ICD.

All clinic visit notes were analyzed for ICD implant recommendation by the general cardiologist. If there was no documentation of ICD discussion in any of the visits, it was assumed that the cardiologist did not offer ICD as an option for therapy. Records of patients with indwelling pacemaker but no ICD were also evaluated for documentation of ICD upgrade proposition (11). Specific reasons for refusal cited following ICD prescription were noted.

Patients were followed for up for at least three months after optimization of guideline-directed medical therapy for HF before a decision for ICD implantation was made by the general cardiologist.

This was in line with 2017 AHA/ACC/HRS guideline for management of patients with ventricular arrhythmias and the prevention of sudden cardiac death (12).

### Statistical analysis

Baseline patient characteristics were analyzed as either continuous or categorical variables. Continuous variables were expressed as means and standard deviations or medians with interquartile range while categorical variables were expressed as frequencies and percentages. Device-eligible patients’ characteristics were stratified into ICD recipient and ICD non-recipient. Fishers Exact test or Chi Squared was used to test for between categorical data and Kruskal Wallis test was used to test the differences between continuous data. Documented reasons for refusal of ICD implantation were recorded as categorical variables and then expressed as frequencies and proportions.

## Results

**Figure 1:**
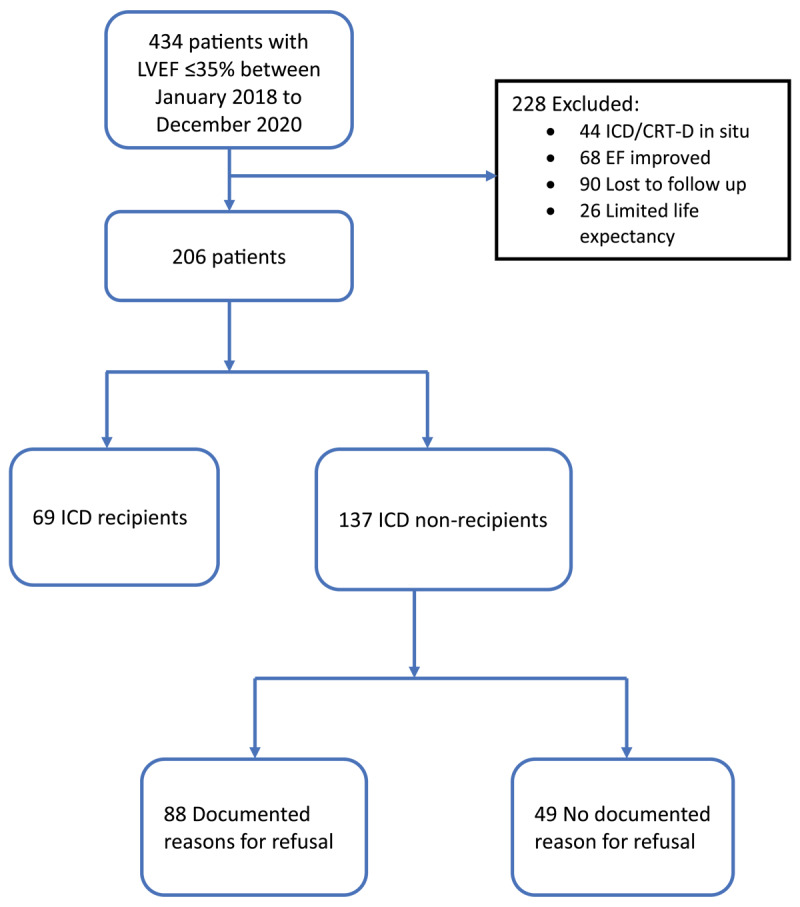
Study flow illustrating patient recruitment and ICD acquisition.

A total of 434 HF patients seen at the heart clinic were assessed for eligibility for inclusion into the study. Of these, 228 patients were excluded; lost to follow-up (n = 90), EF improved to >35% (n = 68), an ICD or CRT-D device placed earlier (n = 44) and limited life expectancy of less than a year (n = 26). The remaining 206 patients were recruited having met the criteria for ICD implantation as seen in Figure 1.

### Demographic and clinical characteristics

The demographic characteristics of ICD recipients contrasted to non-recipients are summarized in Table 1 below. Compared to ICD recipients, majority of ICD non-recipients were of black race (91.2% vs. 79.7%; p = 0.02) and rural dwellers (18.7% vs 8.2%; p = 0.03). Further, ICD non-recipients were less likely to possess private insurance as compared to ICD recipients (42.3% vs 63.8%; p = 0.01). Having a non-cardiology primary physician was associated with non-uptake of ICD (16.1% vs 5.8%; p = 0.045). Among the 137 ICD non-recipients, 49 (35.8%) lacked documentation of discussion regarding the need for ICD implantation. There were no significant age and gender differences between study participants in the ICD recipient and non-recipient groups.

**Table 1:** Demographic and clinical characteristics of ICD Recipients in comparison with Non-Recipients.


		ICD IN SITU				P VALUE

		YES		NO		

		(n = 69)		(n = 137)		

**Age (years) (median [IQR])**		61.3 [53.9, 66.4]		62.2 [53.7, 71.7]		0.431

**Gender (Male)**		55	79.7%	94	68.6%	0.101

**Ethnicity**	Black	55	79.7%	125	91.2%	0.019

	Others	14	20.3%	12	8.8%	

**Residence (n = 187)**	Rural	6	8.7%	25	18.2%	0.031

	Urban	62	89.9%	94	68.6%	

**Primary cause of Heart Failure (n = 203)**	Ischemic CMP	42	63.6%	62	45.3%	0.014

	Non-Ischemic CMP	24	36.4%	75	54.7%	

**Left Ventricle EF (Baseline)**		30.0 [20.0, 35.0]		25.0 [15.0, 30.0]		0.003

**New York Heart Association Class**	I	7	10.1%	22	16.1%	0.241

	II	34	49.3%	74	54.0%	

	III	28	40.6%	41	29.9%	

**History of syncope (n = 200)**		14	20.3%	4	3.1%	<0.001

**Ventricular arrhythmia (n = 197)**		21	30.4%	5	3.9%	<0.001

**Comorbidities**	Diabetes Mellitus	20	29.0%	62	45.3%	0.34

	Hypertension	34	49.3%	85	62%	0.1

	Cancer	4	5.8%	7	5.1%	1.0

	Previous stroke	4	5.8%	9	6.6%	1.0

	Chronic kidney disease	8	11.6%	31	22.6%	0.062

	Dementia	0	0.0%	2	1.5%	0.552

	HIV	1	1.4%	2	1.5%	1.0

**Primary physician**	General Cardiologist	65	94.2%	115	83.9%	0.045

	Non-Cardiology Physician	4	5.8%	22	16.1%	

**Mode of payment**	Cash Paying	44	63.8%	72	52.6%	0.139

	Insurance	52	75.4%	84	61.3%	0.119

**Type of Insurance**	NHIF	8	11.6%	26	19.0%	0.233

	Private	44	63.8%	58	42.3%	0.005


Majority of ICD recipients had ischemic cardiomyopathy (63.6% vs. 45.3%; p = 0.01), history of syncope (20.3% vs. 3.1%; p < 0.01) and ventricular arrhythmias (30.4% vs. 3.9%; p < 0.01) compared to ICD non-recipients. The NYHA, baseline EF and ECG diagnosis were not significantly different between the two groups. In addition, the prevalence of other comorbidities such hypertension, cancer, previous stroke and chronic kidney disease was not correlated with ICD use.

ICDs were implanted in 69 out of the 206 ICD eligible patients (33.5%). Among the 69 with ICD, 44 had ICD only while 25 had cardiac resynchronization therapy with defibrillation (CRT-D) devices. Primary prevention was the most frequent indication (69.6%). ICD procedure-related complications were 8.7% as depicted in Table 2.

**Table 2:** ICD placement data.


	NUMBER OF PATIENTS	PERCENTAGE OF PATIENTS

**ICD Recipients**	**69**	**33.5%**

Type of device		

ICD	44	63.8%

CRT-D	25	36.2%

Type of ICD device		

Single Chamber	20	45.5%

Dual Chamber	24	54.5%

MRI Compatible	53	76.8%

**Complications related to ICD procedure**	6	8.7%

Complications		

Pneumothorax	1	1.4%

Lead Displacement	4	5.8%

Infection	1	1.4%


Of the 137 ICD non-recipients, 88 (64.2%) had documented reasons for refusal. The major reason for non-uptake was the inability to pay for the device (75%). Additional reasons cited were fear of device procedure (18.2%) and lack of belief in ICD (4.5%) as seen in Table 3 below.

**Table 3:** Documented reasons for ICD refusal.


DOCUMENTED REASONS FOR ICD REFUSAL (n = 88)	NUMBER OF PATIENTS	PERCENTAGE OF PATIENTS

**Most common reasons**		

Unable to pay for the device	66	75.0%

Concerned of risks	3	3.4%

Fear of device procedure	16	18.2%

Does not believe in ICD	4	4.5%

Other	5	5.7%

**Other reasons**		

Awaiting EF improvement	2	2.3%

Dementia	1	1.1%

Need more time to decide	2	2.3%


## Discussion

This is the first local study focused on uptake of ICDs in patients with significant heart disease in our hospital and we believe it represents the case situation in Kenya and most of SSA. The study finding of a 33.5% rate of ICD implantation mirrors a previous study conducted in patients post myocardial infarction. Varwani et al analyzed the uptake of ICD among post-myocardial infarction patients with depressed LVEF≤35%. The uptake was 35.7% (5/14) after six weeks of follow up (13). However, this present study had both ischemic and non-ischemic cardiomyopathy patients and a longer duration of follow-up. In contrast to studies in Canada and USA, this study demonstrates a gap in the level of ICD uptake (8,9,14,15,16). Lyons et al demonstrated yearly ICD uptake ranging between 59% to 68% in Canada. The study population had both ischemic and non-ischemic cardiomyopathy patients with a mean LVEF of 33% (8). Similarly, Lin Zhang et al described primary prevention ICD implantation rate of 43% among HF patients with reduced EF ≤ 35% in a tertiary care setting in the USA (15). This difference is possibly explained by the contrasting levels of healthcare resources and access, physician attitudes and patient preferences.

Table 4 below summarizes some of the studies that have assessed ICD uptake in different populations.

**Table 4:** ICD Uptake in different populations.


AUTHOR	YEAR	COUNTRY	POPULATION STUDIED	ICD UPTAKE

Bernier et al	2019	Canada	PP in ischemic and non-ischemic CMP.	36%

Varwani et al	2018	Kenya	PP and SP post-acute myocardial infarction.	35.7%

Lin Zhang et al	2015	USA	PP in ischemic and non-ischemic CMP.	43%

Lyons et al	2014	Canada	PP in ischemic and non-ischemic CMP.	59–68%

Mehra et al	2012	USA	PP in both Ischemic and Non-Ischemic CMP.	64%

LaPointe et al	2011	USA	PP and SP in ischemic and non-ischemic CMP.	59%


PP, Primary prevention; SP, Secondary Prevention; CMP, Cardiomyopathy.

The demographic factors found to be associated with non-uptake in this study were race and residence. Black race compared to other racial groups was significantly linked with non-uptake. Hess et al. noted that black and other ethnic minorities in the USA were less likely to receive an ICD. This could be due to multiple reasons including inability to pay, cultural influence, and varying health literacy. Of note is that there was no physician discrimination in ICD counseling among blacks (17). IMPROVE HF study demonstrated a substantial gain in ICD utilization among eligible black patients as a result of quality improvement initiatives (9).

This study revealed that rural dwellers had fewer ICD implanted compared to urban dwellers. Parkash et al noted significant differences in referral rates between rural and urban dwellers in Canada due to higher rates of referral refusal in the former group (18).

In this study, there was no association between non-uptake with age. The median age of our study population was 62 years (IQR 53.7–69.5) which is relatively younger when compared to earlier reports (11,16). In these studies, eligible patients in this age group were likely to get an ICD implanted.

About two-thirds of patients with ICD in this study were men consistent with pivotal studies that informed current guidelines including MADIT I, MADIT II, and SCD-HeFT (4,5,6). This is attributable to the higher prevalence of coronary artery disease among men compared to women. However, there was no association between female gender with non-uptake in this study. Similarly, Zhang et al found no association between gender and ICD uptake for primary prevention at a tertiary center in the USA among HF patients (15). On the contrary, Curtis et al found that women were less likely to receive an ICD for both primary prevention and secondary prevention of sudden cardiac death (19). Additionally, Amit et al in Israel also showed that female patients were unlikely to be implanted due to a lower prevalence of ischemic cardiomyopathy compared to men (20).

In this study, 75 (54.5%) of HF patients without ICD had non-ischemic cardiomyopathy. This is in keeping with the landmark randomized control trials that showed most patients with non-ischemic cardiomyopathy were less likely to have ICD implantation (3,6). In this analysis, the prevalence of other comorbidities such as hypertension, cancer, previous stroke and chronic kidney disease were not significantly associated with ICD uptake. A retrospective study by Lin Zhang et al at a tertiary urban hospital in the USA showed no connection between comorbidities and ICD uptake for primary prevention among HF patients with depressed ejection fraction (15). In this study NYHA functional class was not associated with non-uptake as opposed to a prospective study by Lee et al in Korea showed that NYHA class ≥III was an independent factor predictive of ICD implantation for primary prevention among eligible HF patients (21).

Consistent with earlier reports by Sadarmin et al., we observed that majority of patients without ICDs were under the care of a non-cardiology primary physician. Lack of referral by a primary care physician could be due to lack of awareness of device guidelines or bias regarding the cost- effectiveness and benefit of ICD implantation (22). Targeted education of primary care physicians regarding guidelines has been described to be key in enhancing ICD utilization rates. Further, measures such as automatic referral to cardiology service if EF is ≤35% and regular internal appraisals have been shown to improve the rate of referral for ICD implantation (23).

The observation in this study that more ICD recipients compared to non-recipients had private insurance is congruous with the determination by Lin Zhang et al. The cost of ICD implantation in the in Kenya is approximately 14,000 US dollars which makes it prohibitive to the majority of patients who pay out of pocket. Further, some insurances including government managed National Health Insurance Fund (NHIF) only cover a percentage of the total cost necessitating significant out of pocket payment.

A notable finding in this study was that most patients declined ICD device therapy due to financial constraints. The majority of the patients paid out of pocket to access care. A prior study by Balbir et al. highlighted inability to pay as the most common barrier among HF patients in developing countries in Asia (24). This is consistent with reports by Bonny et al that emphasized the high cost of procedures as a hindrance to device therapy in SSA (25). Some patients cited fear of device procedure, a concern reported by Singh et al (24). A minority of patients awaited improvement of EF while adopting lifestyle changes such as cessation of alcohol.

### Strengths and Limitations

This is the first study in Kenya looking at ICD implantation rates for eligible patients and assessment of non-uptake. Since the study setting is a tertiary referral center with a diverse catchment area of East, Central, and Southern African countries, the study population is representive of the region. In addition, since ICDs in Kenya are mostly offered in private hospitals, it represents the case scenario in other private facilities.

This present study is not without limitations. There is a high likelihood of referral bias, which may be due to multiple factors ranging from socio-economic to physician and health system factors. The study participants were reviewed by general cardiologists rather than HF specialists. It is possible that review by HF specialists could have influenced the level of ICD uptake. The study setting is a private tertiary level health facility serving largely an insured urban middle-class population. Hence the level of ICD implantation reported could be exaggerated due to referral bias. Certain physician factors such as knowledge and attitudes as barriers to ICD implantation could be better analyzed prospectively using a qualitative approach. Lastly, being a retrospective study, missing data was a challenge particularly with reasons for ICD refusal.

## Conclusion

ICDs are underutilized among eligible HF with reduced EF patients in Kenya. The majority of patients without ICD had no private insurance, had non-ischemic cardiomyopathy and had a non-cardiology primary care physician. Financial constraints were the major reason for refusal of ICD implantation. Early referral of HF patients with LVEF <35% to HF specialists to optimize HF medications and make ICD recommendation is needed. Further studies are needed to widen the scope of general and private hospitals in SSA region to better understand the magnitude of the problem of non-uptake of ICD in eligible patients with HF with reduced EF. Given that economic factor was the largest reason for non-uptake of ICDs, major concerted efforts are warranted to bridge these patients to care.

## Additional File

The additional file for this article can be found as follows:

10.5334/gh.1346.s1 Supplementary file 1. Case Report Form.
