# Supplementary material for: Factors Associated With Non-Uptake of Implantable Cardioverter-Defibrillator (ICD) Among Eligible Patients at a Tertiary Hospital in Kenya
Source: Glob Heart. 2024 Aug 16;19(1):66. doi: 10.5334/gh.1346 (PMC11328684; doi:10.5334/gh.1346)
Supplement: Supplementary file 1. — Case Report Form. [file gh-19-1-1346-s1.pdf]

Supplementary file 1: Case Report Form

Section A. Participants' Demographic data

1. Study ID _______________
2. Hospital Number ________________
3. Participant's Date of Birth. ________________ (DD/MMM/YY)
4. Participant's gender:
   i) Male ☐
   ii) Female ☐
5. Participant's Ethnicity: Black. ☐
   Asian ☐
   Caucasian. ☐
   Other

6. Residence: i) Rural ☐
   ii) Urban ☐

Section B. Clinical Data.

7. Primary cause of heart failure:
   i. Ischemic CMP ☐
   ii. non-ischemic CMP ☐
   iii. Not known/information cannot be ascertained from the medical file. ☐
8. Left Ventricle ejection fraction.
   i. Baseline EF______
      Date. ____________
   ii. Reassessment EF_____
      Date. _____________
9. New York Heart Association class.
   i). I ☐
   ii). II ☐
   iii). III ☐
   iv). IV ☐

10. History of Syncope. Yes. ☐ No.☐
11. Documented Ventricular arrhythmia. Yes. ☐ No.☐
12. Other comorbid conditions present:
    i. Diabetes mellitus. ☐
    ii. Hypertension. ☐
    iii. Cancer (please specify type) ☐
    iv. Previous stroke ☐
    v. CKD ☐
    vi. Dementia. ☐
    vii. Psychiatric illness. ☐

13. Baseline ECG diagnosis
    i. sinus rhythm ☐
    ii. Atrial fibrillation (any) ☐
    iii. Paced rhythm ☐
    iv. LBBB, if yes QRS duration ☐
    v. RBBB, if yes QRS duration ☐
    vi. Ventricular Premature Beats ☐

Section C. ICD.

14. Documented ICD counselling/prescription. Yes ☐ No ☐
15. ICD in Situ: Yes ☐ No ☐
16. Date of ICD Implantation. (DD/MMM/YY)
17. Any complications related to ICD procedure. Yes ☐ No ☐
    If yes, Tick appropriate.
    i). Bleeding from the incision site or a hematoma (Requiring intervention) ☐
    ii). Pneumothorax ☐
    iii). Mechanical ☐
    vi). Infection ☐
    v). Others ☐ specify________

Section D. System Data.

18. Primary Physician.
    i. General Cardiologist. Yes ☐ No ☐
    ii. Non-cardiology physician. Yes ☐ No ☐

19. Mode of ICD payment.i). Cash paying. ☐
ii). Insurance.
    a. Government (NHIF) ☐
    b. Private ☐
iii). Combined Cash and Insurance. ☐

Section E. Reasons for refusal.

20. Documented reason for refusal. Yes ☐ No ☐

If yes, what was the reason? Tick applicable.

i. Unable to pay for the device ☐
ii. Concerned of risks associated with the device ☐
iii. Fear of device procedure ☐
iv. Doesn’t believe in benefit of ICD ☐
v. Cultural/Religious belief ☐
vi. Conflicting opinions from medical staff ☐
vii. Unknown ☐
viii. Others ☐
